# Mindfulness in the focus of the neurosciences - The contribution of neuroimaging to the understanding of mindfulness

**DOI:** 10.3389/fnbeh.2022.928522

**Published:** 2022-10-17

**Authors:** Bruno J. Weder

**Affiliations:** Support Centre for Advanced Neuroimaging (SCAN), Institute for Diagnostic and Interventional Neuroradiology, Inselspital, Bern University Hospital, University of Bern, Bern, Switzerland

**Keywords:** mindfulness, mind wandering, focused attention, open monitoring, self-specifying processes, magnetic resonance imaging, default mode network

## Abstract

**Background:**

Mindfulness affects human levels of experience by facilitating the immediate and impartial perception of phenomena, including sensory stimulation, emotions, and thoughts. Mindfulness is now a focus of neuroimaging, since technical and methodological developments in magnetic resonance imaging have made it possible to observe subjects performing mindfulness tasks.

**Objective:**

We set out to describe the association between mental processes and characteristics of mindfulness, including their specific cerebral patterns, as shown in structural and functional neuroimaging studies.

**Methods:**

We searched the MEDLINE databank of references and abstracts on life sciences and biomedical topics *via* PubMed using the keywords: “mindfulness,” “focused attention (FA),” “open monitoring (OM),” “mind wandering,” “emotional regulation,” “magnetic resonance imaging (MRI)” and “default mode network (DMN).” This review extracted phenomenological experiences across populations with varying degrees of mindfulness training and correlated these experiences with structural and functional neuroimaging patterns. Our goal was to describe how mindful behavior was processed by the constituents of the default mode network during specific tasks.

**Results and conclusions:**

Depending on the research paradigm employed to explore mindfulness, investigations of function that used fMRI exhibited distinct activation patterns and functional connectivities. Basic to mindfulness is a long-term process of learning to use meditation techniques. Meditators progress from voluntary control of emotions and subjective preferences to emotional regulation and impartial awareness of phenomena. As their ability to monitor perception and behavior, a metacognitive skill, improves, mindfulness increases self-specifying thoughts governed by the experiential phenomenological self and reduces self-relational thoughts of the narrative self. The degree of mindfulness (ratio of self-specifying to self-relational thoughts) may affect other mental processes, e.g., awareness, working memory, mind wandering and belief formation. Mindfulness prevents habituation and the constant assumptions associated with mindlessness. Self-specifying thinking during mindfulness and self-relational thinking in the narrative self relies on the default mode network. The main constituents of this network are the dorsal and medial prefrontal cortex, and posterior cingulate cortex. These midline structures are antagonistic to self-specifying and self-relational processes, since the predominant process determines their differential involvement. Functional and brain volume changes indicate brain plasticity, mediated by mental training over the long-term.

## Introduction

Through mindfulness, we discern new aspects of experience, rather than seeking to confirm established convictions when we assimilate our experience (Langer and Moldoveanu, 2000). In mindfulness, “what” is not as important as “how:” immediate experience is paramount. Active recognition of the new sensations we perceive binds our attention to the present, increasing our awareness of the context of our activities and our perspectives on them. The work of freeing ourselves from the models and categories of the past brings a new awareness: a subjective feeling of involvement in ongoing events and more intense experience of the “Here and Now.”

Since the early 1970s, mindfulness studies have elucidated the basic characteristics of mindfulness (Brown et al., 2007). These are (1) dedication to immediate experience and concentration on the present, (2) assuming a dispassionate attitude that allows instantaneous assessment of what is observed, and (3) appreciation and acceptance of sensations, feelings, or thoughts as they arise. This fundamentally impartial attitude allows individuals to anticipate intrusion of obstructive rumination and subjective values or preferences, increasing the flexibility of their thought and improving coping strategies in overwhelming or stressful situations and strengthening resilience (Keye and Pidgeon, 2013).

The first studies often sought to determine the effects of mindfulness on health. Kabat-Zinn et al. (1985) investigated the significance of mindfulness to the self-control of pain. Shapiro and Schwartz (2000) proposed that mindfulness helped reduce stress [Mindfulness Based Stress Reduction, MBSR] and developed an integrated model for stress reduction and health. Teasdale et al. (2000) thought mindfulness opened the possibility one could monitor one's own cognitive processes and could aid in treating mental illnesses. They found the relapse rate for depression decreased when patients were treated with behavioral therapy and mindfulness (Teasdale et al., 2002).

Industry had a separate early interest: applying techniques to motivate workers and managers to practice mindfulness. Industry studies of mindfulness showed the practice fostered creativity and helped reduce burnout (Goodman and Schorling, 2012; Langer, 2014). Davenport and Pagnini (2016) elucidated the conflict between mindfulness and indifference in education with the goal of promoting more mindful learning in the schools.

The scientific coordinator and moderator of the first Mind-and-Life-Dialogs^1^ (Varela, 1996) proposed that the meditative potential of human experience is a necessary complement to the inner representations of the external world posited by cognitive sciences. He conceived of neurophenomenology as a reciprocal relationship between phenomenological access and experiential structures and inner representations of the external world. Varela et al. (2016) asked this neurophenomenological question: Can classical cognitive science assess the experiential content of mental states in the philosophical term qualia? He saw the meditation of Buddhist monks, characterized by their ability to be present in body and mind, as the exemplar of a rigorous paradigm for investigating experience at first hand. In the Buddhist tradition, meditation is a concerted act of body and mind, which receive perceptions while the individual's conscious awareness remains directed, uninterrupted, at the object perceived (Revel and Ricard, 1999). Buddhist practice is also confident of the stream of consciousness (James, 1890) described as having five characteristics: (1) subjectivity; (2) permanent change; (3) continuity; (4) autonomy regarding the specific mental activity; and (5) preference for distinct objects.

About 20 years ago, separate research groups identified brain areas, mainly at midline cortices, in which BOLD (blood oxygen level dependent) signals decreased as BOLD activity in areas involved in goal-directed behaviors increased (Shulman et al., 1997; Gusnard and Raichle, 2001; Mazoyer et al., 2001). Gusnard and Raichle (2001) recognized that these areas, which constitute the default mode network (DMN), are active at baseline and become more active during self-referential tasks. These discoveries heralded a new era and a new perspective: we could now use imaging techniques to explore mental processes like mindfulness. Garrison et al. (2015) showed that in experienced meditators DMN is typically characterized by suppression of default mode processing during meditation, beyond the resting state function observed during another active, self-relational cognitive task. This and altered baseline during rest seem to be unique features of long-term meditation.

This review is based on five themes found in ongoing conceptual and theoretical disputes on mindfulness. These themes are associated with both behavioral patterns and neuroimaging data: (1) proposals in the literature for an unequivocal operational definition of mindfulness; (2) experimental requirements derived from operational definitions met in corresponding neuroimaging projects; (3) dimensions of mindfulness and its connections to other mental processes, e.g., believing; (4) contributions of neuroimaging studies to our understanding of mindfulness, specifically studies on DMN and areas engaged in interoception and exteroception; and (5) the dynamic association between expertise in mindfulness meditation and related morphological and functional imaging patterns.

The five thematic sections of the review focus on pressing questions and observations: 1. “The quest for an operational definition of mindfulness—a semantic issue;” 2. “Dimensions of believing;” 3. “Dimensions of believing and their interrelation with mindfulness,” delimiting the distinction between perceptual processing and mindfulness *via* an evaluative component; 4. “Studying mindfulness with neuroimaging using MRI—concepts and technical aspects;” 5. “Neuroimaging studies of mindfulness—shaping the brain in parallel with the experience of mindfulness meditation.” The search terms we used to identify the papers we included in the review are summarized in the Supplementary material in partitions that align with these sections (Supplementary Table S1).

This review extracts phenomenological experiences across populations with varying degrees of experience in mindfulness and correlates experience with structural and functional neuroimaging patterns that reflect the way mindful behavior is processed by the constituents of the default mode network during specific tasks.

## The quest for an operational definition of mindfulness—A semantic issue

The prerequisite for an operational definition of mindfulness is adherence to descriptive language that permits exploration and understanding of neuroimaging results across disciplines (Lutz et al., 2015). Bishop et al. (2004) reported proposals for an operational definition made at a consensus conference. The primary concern of attendees was specifying the essential components that would serve as the basis for verifiable predictions. The secondary concern was characterizing measures to validate the resulting construct. These efforts produced a model of mindfulness comprising two elements: (1) regulating mindfulness to heighten awareness of mental activity during an experience; (2) deciding to focus one's attention on one's immediate experience, characterized by an attitude of curiosity, openness and receptivity. The conference's initiatives aroused prompt attention and provoked ongoing discussion about developing this model. Discussants expressed reservations about the model's completeness, asking if the model comprised all the necessary components. Kabat-Zinn (1990), who defined mindfulness as paying purposeful attention characterized by immediacy and withholding judgment, proposed a different but similar two-component model.

Mindfulness creates a mental state in which one attends to sensations, feelings, and thoughts as they emerge in the stream of consciousness. The skill of observing impartially and immediately can be learned within the framework of meditation. Lutz et al. (2015) delineated four contextual features of meditation: physical posture; non-aversive affect; axiological framework; and maintenance or retention of experience. Physical postures that facilitate meditation techniques include sedentary practices like FA, OM, and ethical enhancement, and also some that require physical exercise like Hatha Yoga (Vago, 2014). The concepts behind Hatha Yoga and ethical enhancement are complex and explicitly extend mindfulness into the external world. They include empathy and demand integrity, which is particularly important in mindfulness-based research (Crane and Hecht, 2018). Ethical and religious contemplation are aspects of an axiological framework that transcend the secular perspective, so they are not a subject of this review.

There is a consensus to classify meditation techniques at least into the categories of FA meditation, OM meditation, and compassion or loving-kindness practices (Fox et al., 2016). FA-meditation focuses attention on a specific bodily act like breathing supported by a controlled posture and should sustain awareness of current experience (Bishop et al., 2004). Claims that this meditative practice predicts introspective accuracy are supported by subjective reports and objective measures of tactile sensitivity, e.g., 2D discrimination or adjusted cortical activity (Fox et al., 2012). Mantra recitation meditation may appear similar to FA meditation but distinct through the inherent focus, i.e., the repetition of a sound, word or sentence spoken aloud or silently (Travis, 2014). OM-meditation is also introspective, characterized by curious and deliberately unrestricted receptivity to experiences, primarily physically but also mentally. The trainee gains flexibility, can regulate and sustain attention, deal immediately with experiences, and keeps an open mind (Posner, 1980). Loving-kindness meditation and compassionate meditation are closely related to each other but differ in their intention: In loving-kindness meditation subjects intend to generate sympathetic feelings for all living beings whereas in compassion meditation they cultivate empathic attitudes and behaviors to the suffering of others (Fox et al., 2016). Components of mindfulness are evident in these practices, like sustained attention and open examination of immediate experience, but they are not the same as mindfulness, which is an internalized disposition to accept experience and retain its context in daily life after meditation training (Gethin, 2011).

FA-meditation is directed to somaesthesia, since it is restricted to interoception, but OM-meditation also encompasses the external world, induced by an open-minded and curious basic attitude. The different traditions of Buddhism emphasize either awareness/mindfulness or concentration/absorption, both of which affect and promote behaviors (Mikulas, 2011). Experienced meditators perform better on the Wilkins counting test than inexperienced meditators (Wilkins et al., 1987; Valentine and Sweet, 1999). Young adults who practiced OM and FM for a week had better executive function scores on an emotional variant of the Attention Network Test than those in a control group that practiced only relaxation techniques (Ainsworth et al., 2013). Different meditation techniques can also produce differences in behavior. Colzato et al. (2012) found that subjects proficient in OM-meditation may be better at tasks that require divided attention than subjects proficient in FA-meditation. Lutz et al. (2008) suggested that FA-meditation promotes effortless concentration while OM-meditation promotes objectless attention.

Physiological effects of mindfulness were also observed, namely decreased neural response to stimuli. Brown et al. (2013) showed that after viewing stimulating images (pleasant or unpleasant), mindful intervention helped decrease amplitude of late positive potential (LPP) after 400 ms. Since LPP is an electrophysiological marker for the emotional valence of a stimulus, this decrease indicates that mindfulness modulates emotion in an early phase of generation, before cognitive suppression can inhibit explicitly expressive behavior (Gross, 2001; Sheppes and Gross, 2011). As an example of implicit sensory-affective-motor processing, it is possible the underlying enactive experiential network (Vago and Silbersweig, 2012) sustains an equanimous frame of mind. Emotion control strategies appear to change with age: people's ability to appraise positively improves with age, while their ability to suppress emotional behavior is maintained, and their ability to implement detached reappraisal declines with age (Shiota and Levenson, 2009).

The quest for an operational definition of mindfulness persisted after the consensus conference. Bishop et al. (2004)'s aim was to distinguish core elements of the model and demarcate collateral features that might actually be beneficial. Shapiro and Schwartz (2000) discussed collateral features including patience, trust, self-restraint, wisdom, and compassion. Some conjectured these benefits emerge as an individual becomes conscious of his own thoughts in a process of continuous mental cultivation (Hölzel et al., 2011; Lutz et al., 2015). Meta-awareness then facilitates the prerequisite of experiential retention. Brown et al. (2007) contended that mindfulness is an attribute of consciousness that pertains to perception rather than cognition. They assert that the distinction between mindfulness as such and the various meditative practices that help practitioners attain mindfulness are insufficient, since these practices emphasize the perception of internal phenomena. Langer (2014) suggested that the definition of mindfulness be extended from awareness of the internal world of sensory stimulation, emotions, or thoughts to attention to the outside world of salient events. This extension opens new possibilities in exercising mindfulness: creation of new categories; a proper world of thoughts freed of old patterns; openness to new experience from a first- or third-person perspective; and recognizing alternative adaptive possibilities in specific circumstances.

A core element of mindfulness is impartial registration of sensations, feelings, and thoughts (Rimes and Wingrove, 2011). Maintaining non-aversive affect is essential to mindfulness, as it may foster positive attitudes like acceptance, loving kindness, compassion, or aesthetic appreciation (Shapiro et al., 2006; Lutz et al., 2015). A responsive positive emotional state like compassion is a prerequisite for open-mindedness and curiosity because it reduces preoccupation with repetitive negative thoughts and redundant speculations (Takano and Tanno, 2009; McEvoy et al., 2010). These thoughts and speculations can afflict the subject with reiterative and critical discourse about the meaning of perceptions and disrupt attention to immediate experience. Open-mindedness, in contrast, facilitates perception during the stream of consciousness and thus sustains flexibility (Moore and Malinowski, 2009). Greenberg et al. (2010) showed that internalizing mindfulness sustained flexibility in those who solved Luchins' water jar test.

After an experience and depending on the degree of activity, the focus of attention in the daily stream of consciousness alternates between attention directed to external events and attention to internal states (James, 1890). Based on stimulus-oriented thoughts (SOT), mindful mental states subserve external perceptions. Individuals often cannot maintain SOTs and lapse into stimulus-independent thoughts (SITs), which are associated with ruminations about past and future and their effects on self-imagination and expectations (Mason et al., 2007a,b). The boundary between mindfulness *sensu stricto* and such self-monitoring is critical. These states of self-focused attention may also cause distress (Nolen-Hoeksema, 1991; Trapnell and Campbell, 1999; Neff, 2003). Such intrusions in our consciousness, experienced as daydreams, can accompany physical activity and correlate negatively with its intensity (Killingsworth and Gilbert, 2010). Self-referential thinking and adherence to old, ingrained attitudes and patterns of thought hinders attention to outer experience and shifts the focus of internal mental activity from impartial, open perception of feelings and thoughts to the irreal realm of wishes and fears (Dambrun and Ricard, 2011). The experiential system loses its immediate relation to reality, reducing the capacity of the affected individual to adapt to new situations or conditions (Rummel and Boywitt, 2014). The lapse into an illusionary world when the mind “wanders” then pervades our thoughts. A careful, web-based study of 2,250 arbitrarily selected individuals indicated that mind wandering prevailed 46.9% of the time (Killingsworth and Gilbert, 2010). During externally directed activity, lapses into mind wandering occurred for at least 30% of the period measured.

A multilevel regression showed that individuals felt unhappier when their minds wandered. The authors concluded, “A wandering mind is an unhappy mind.” The regression indicated that the content of their thoughts was a better predictor of the individual's happiness than what they were doing. Mason et al. (2007a) ascertained, in interviews with subjects at rest immediately after a functional MRI session, that mind wandering was common. Participants engaged in stimulus-oriented thinking 26% of the time, focused on their physical state 15% of the time, and were preoccupied with stimulus-independent thoughts 59% of the time. Stimulus-independent thoughts focused on the future 26% of the time, on the past 23% of the time, and were unspecified 10% of the time. By nature, mind wandering tends to inattention, contrasting clearly with the decentred, unconstrained attitude of mindfulness. Others have also reported predominantly negative effects of mind wandering, especially when the mind wanders to past events (Smallwood and O'Connor, 2011; Stawarczyk et al., 2013). One contradictory study observed that positive mood effects and mind wandering were reciprocal (Smallwood et al., 2009).

### Scales for the assessment of mindfulness and inattention

Validated mindfulness scales characterize subjective dispositions or traits and describe a subject's tendency to be mindful in daily life. These scales include the Mindful attention Awareness Scale (MAAS) (Brown and Ryan, 2003), the Freiburg Mindfulness Inventory (FMI) (Walach et al., 2006), the Kentucky Inventory of Mindfulness Skills (KIMS) (Baer et al., 2004), the Five Facet Mindfulness Questionnaire (FFMQ) (Baer et al., 2006) and the Imaginal Processes Inventory (http://neuroinformatics.harvard.edu/w/public/images/5/55/Ipi.pdf). The Imaginal Processes Inventory assesses the risk an individual will lapse from mindfulness into daydreams or mind wandering (Supplementary Table S2: Scales measuring mindful traits). The Toronto mindfulness scale measures mental states; this scale can be correlated with the dispositions of mindfulness described above (Lau et al., 2006). All these are Likert scales, and all these single factor and multifactor scales contain ambiguities that must be resolved in future studies. A pressing question is how much their results depend on meditation experience and whether their findings are consistent with experimental mindfulness tasks (Bergomi et al., 2013; Chiesa, 2013). We also cannot be sure if mindful traits are related or independent factors.

## Dimensions of mindfulness

A dual concept of personality theory posits that human behavior relies on two information processing systems that work in parallel and interact. These are the rational and experiential frameworks, e.g., Epstein (2003)'s cognitive—experiential self-theory (CEST). The two frameworks support first and third person perspectives. A hypothesis of classical cognitive science is that the first person perspective of empirical self-observation is a subject's privileged account of their own experience. This privileged view is inaccessible to other observers and thus irreducible to third person data (Searle, 1994). Third person perspective reduces perceptions to objects and processes that exist outside, and thus are independent of the subject's mind. Third person perspectives provide data about the objective structure and dynamics of physical systems (Chalmers, 2013). The classical assumption is dualistic, posing a dichotomy between indirectly ascertainable objects and processes and internally experienced percepts.

Scholars are increasingly criticizing the presumption of a dichotomy. Choifer (2018) partially resolves this dilemma by linguistically linking these two perspectives to the personal pronoun. He proposes that the subject exhibits two modes of consciousness: reflective or non-reflective. These two modes of being in the world allow the subject to occupy one of two mutually exclusive perspectives at a given time. In the reflective mode, the third person perspective is scientifically accessible. When the subject detaches from self-referential thoughts, they may take an experiential attitude to self-observation, the precondition for metacognitive skills like monitoring one's own perceptions (Pasquali et al., 2010).

Varela and Shear (1999) integrate the first person perspective with non-reflective thoughts as lived experience associated with cognitive and mental events into a science of consciousness. They acknowledge that this lived experience must be substantiated by third-person studies. According to this theory, the perspective of a second person (e.g., an experienced tutor) may mediate between the perspectives of the first and third person (Pauen, 2012). The discussion of the theoretical framework that supports the argument for an intermediate, second person perspective is out of scope of the review but validating second-person methods for studying human consciousness would require first establishing objective methods for comparing results across different subjects and tutors (Olivares et al., 2015). A new cognitive science construct derived from probabilistic models and based on prediction coding and the free energy principle treats both the metaphysical self (“I,” the subject of experience) and the phenomenal self (“me,” the object of experience) as if they occupied different levels of the phenomenal self-model (Metzinger, 2018). This distinction is purely pragmatic; it forfeits the subject of experience and asserts that an experience can be owned (Wozniak, 2018). The main questions are now: How may we describe and reliably estimate the metacognitive competence of self-monitoring? How can we grasp effects of mindfulness on the subject's experience? And how shall we integrate aspects of mindfulness vs. mindlessness into a neurobiological concept that matches patterns visible in neuroimaging that could be associated with these states?

To generate scientific hypotheses (Lutz et al., 2015) proposed a heuristic tool: orientation on a phenomenological matrix of mindfulness. They suggested we could map focused attention, open monitoring, mind wandering, and rumination within three-dimensional space by measuring object orientation, dereification, and meta-awareness. OM could be clearly differentiated from FA in experienced subjects along the axis of object orientation [1], like OM and FA can be differentiated from mind wandering along the axis of dereification [2] and OM can be differentiated from mind wandering along the axis of meta-awareness (the indication for monitoring of experience) [3]. One could also map the secondary dimensions of aperture of the focus of interest, clarity of the percept, stability of disposition and effort, each of which would reveal qualities of these mental states. Based on self-reports from a neurophenomenological experiment, OM and FA were distinct. The distinction was illustrated by the broad range of attention novices and experienced meditators exhibited during OM (Abdoun et al., 2019). FA is less clear (less vivid experience) and less stable (experiences of shorter duration) than OM, because FA relies on theoretical background and experiential knowledge to calm and slow down the mind (Revel and Ricard, 1999). Dereification is associated with impaired ability to discriminate between mental phenomena and depictions of reality. Together with unavailable meta-awareness, dereification characterizes mind-wandering, impacting negatively on wellbeing (Dahl et al., 2015).

Christoff et al. (2016) presented a complementary two-dimensional framework that places mental states subjected to deliberate constraints on one axis and mental states subjected to automatic constraints on the other. Subjects can exert cognitive control to govern their mental states deliberately (Miller, 2000). Cognitive control is most strongly exerted during goal-directed thought, less common during creative thinking and mind wandering, and least common during dreams. Automatic constraints are fundamentally different because they cannot be controlled by cognition and are most likely driven by affective and sensory salience (Todd et al., 2012), e.g., ruminations, obsessive thoughts, and addictive cravings.

Five days of integrative body-mind training grounded in traditional Chinese medicine, which included breathing adjustment and mindfulness training, gradually cultivated effortless attention and improved conflict resolution, as measured by the attention network test (ANT) (Tang et al., 2007). A study that tested the threshold for conscious perception and working memory capacity found that in meditation novices who engaged in mindfulness-based stress reduction, these capacities improved significantly more than in those who practiced alternative strategies; however, it was impossible to strictly differentiate confounding factors like test effort and stress reduction not caused by mindfulness (Jensen et al., 2012). As compared to a distinct focused attention awake state, measuring the relative concentrations of brain metabolites using ^31^P Magnetic Resonance Spectroscopy indicated an enhanced energetic state induced by a FA meditation state in the basal ganglia and temporal lobes and, furthermore, a down-regulation of ATP-turnover in the occipital and frontal lobes after a 7 weeks training (Galijašević et al., 2021).

Long-term practice of meditation within the Tibetan Buddhist tradition cultivates a special form of attentional expertise (Brefczynski-Lewis et al., 2007) in which practitioners can sustain attention on an external or internal object over time. This is one-pointed concentration: when a state of equanimity is achieved, the dichotomy of subject and object may eventually disappear (Revel and Ricard, 1999). One study examining the effects of 3 months of systematic mental training in concentration meditation on information processing found that the practice seems to ameliorate the so-called “attentional blink deficit” in which two targets compete for limited attentional resources (Slagter et al., 2007).

Vago and Silbersweig (2012) propose a comprehensive conceptual framework to describe the functional relationship between mindfulness processing and neurobiological mechanisms: S-ART (Self-Awareness, -Regulation, and -Transcendence). Its constituents are the task positive networks (cf. attention to the external world) of the enactive experiential self (EES) and of the experiential phenomenological self (EPS), the task negative network (cf. internally directed mentation) of the narrative self (NS), and an integrative fronto-parietal control network (FPCN). The EES reflects elementary processes that integrate exteroception, proprioception, kinaesthesia, and interoception to establish a physical self-percept, organized at the level of the unconscious (James, 1890; Damasio, 1999; Craig, 2003). The NS describes a self-concept based on reflective and evaluative perception of physical, social, and psychological domains (Christoff et al., 2011). In contrast, EPS comprises higher level percepts acquired through self-specifying, primarily non-judging cognitive processes during present awareness; EPS is thus distinct from the self-related processes of the NS (Gallagher, 2000). While EES, EPS, and NS may be functionally independent, the FPCN generates consistent hub patterns, activating each system differently in practiced tasks and flexibly adapting to novel tasks (Vincent et al., 2008; Cole et al., 2013).

## Dimensions of believing and interrelations with mindfulness

Beliefs and disbeliefs are unequivocally mental processes with specific neural correlates, as Sacks and Hirsch (2008): According to the seminal work of Harris et al. (2008), contrasting beliefs and disbeliefs evinced consistently involvement of ventral medial prefrontal cortex (mPFC) using fMRI when subjects assessed written propositions. Interestingly, subjects were quicker to judge statements true than false or undecidable, suggesting that the latter two judgements require more complex information processing. Seitz et al. (2022) proposed three categories of beliefs based on their inherent processual properties: (1) empirical (implicating objects); (2) relational (implicating events); and (3) conceptual (implicating narratives).

These categories reflect varying mental demands and relationships between knowledge and belief. Beliefs are propositional attitudes like desires; at best, they are probabilistic approximations of reality because our sensori-motor and cognitive perceptive systems are limited, as are our predictions of emerging actions (Howlett and Paulus, 2015; Seitz et al., 2016). An example of an empirical belief would be probabilistic modeling of the sensori-motor hand skill of object exploration, which relies on extracting the first three components from a digital data glove. Analyzing the principal component with around 80% variance would enable us to describe finger positions in space over time and thus designate the type of the multifinger task as finger gaiting (Krammer et al., 2020). Structurally, the probabilistic map of the brain lesion (part of a distributed cortical neuronal network) predicted recovery from tactile agnosia vs. persistent disorder over the long-term with 90% accuracy (Abela et al., 2019).

Relational beliefs include percepts of objects and subjects (Seitz et al., 2022). Objects, tools, or interfaces may be integrated because people believe and trust in their usefulness. Eventually, this iterative and embodied process becomes a routine in which use is automatic (Nehaniv et al., 2013). Personal interactions are similarly mediated and stabilized by trust in familiar wordings, manners of speech, and concomitant intimate gestures validated as individuals grow (Seitz et al., 2018). Conceptual beliefs appear in our narratives, often in stories about our past and thoughts about our future, and shape our autobiographical memory (Fivush et al., 2011). Confronted with a conceptual question, subjects decide whether to seek maximal value based on momentary beliefs or explore an issue from several perspectives with the goal of preserving multiple options. The decision to seek maximal value is driven by experience and promises of reward; the action may consolidate our beliefs or make us rigid (Duncan and Peterson, 2014). The decision to preserve options spring from mindfulness, which allows us to better adapt to a concrete situation because it grants us more freedom and can update our beliefs (Langer, 2014).

Believing processes are products of the empirical, relational, and conceptual processes detailed above, and are distinguished by self-relational valuation; they spur action and a learning process that helps us predict errors (Seitz et al., 2018). Multiple factors set the course of a person's believing processes: (1) becoming aware of actions or internal narratives; (2) experiencing agency and ascribing ownership; (3) referring perception to the real world; (4) emotional binding and increasing trust that comes from relying on percepts. When narratives productively use unrealized possibilities, this may raise the risk of counterfactual explanations (Brugger and Graves, 1997). The limitations of the pure third person perspective of classical cognitive science are clear when we examine trusting beliefs. We may trust intentions, behavior, dispositions, and institutions, posing difficulties for an operational definition and modeling trust related judgements (Vidotto et al., 2012).

In contrast, mindfulness is self-generating and self-sustaining, resistant to mindset manipulation (Langer et al., 2010). Mindfulness is distinguished by a mainly non-judgmental behavior, facilitated by a decentred attitude (Shepherd et al., 2016). Decentring makes open-minded acceptance possible and is an essential component of self-awareness. Decentring mediates between mindfulness and positive affect, but not between mindfulness and positive thinking. Mindfulness, however, correlates directly with positive thinking (ben Salem and Karlin, 2022). This suggests that decentring and mindfulness are separable constructs that travel distinct pathways (Gecht et al., 2014). But mindfulness and awareness intertwine and together make it possible for people to perceive thoughts, beliefs, motivations, and feelings clearly (Brown and Ryan, 2003; ben Salem and Karlin, 2022).

Table 1 gives an overview of up-dated personality concepts, which include now experience from a first person perspective and is basic for verification of mindfulness effects on behavior by neuroimaging methods.

**Table 1:** A change of personality concept to integrate the subject's experiential mode of information processing.

(1) Classical dual concept of personality posits two information processing systems in humans: a rationale and an experiential one.
(2) The dilemma of classical cognitive science is: Subjective experience is not accessible to other observers whereas perception of objects and processes are accessible.
(3) The subject exhibits two modes of consciousness: a non-reflective (i.e., a 1st first person perspective) and a reflective (i.e., a 3rd person perspective, ownership of experience).
(4) A probabilistic model posits: the metaphysical self (“I,” the subject of experience) vs. the phenomenal self (“me,” the object of experience).
(5) A practical approach for a science of consciousness: exact describing the phenomenal-self according to a reflective, methodically guided phenomenological analysis.
(6) Mindfulness and believing interact with living experience and are mutually antagonistic (principle of subjective detachment vs. principle of subjective evaluation).
(7) Here the objectives of the assessment of mental processes are: to differentiate between the manifestations and mechanisms of unconscious EES, conscious EPS and NS.

*Searle (1994), Epstein (2003), Gallagher and Varela (2003), Vago and Silbersweig (2012), Chalmers (2013), Choifer (2018), Metzinger (2018), and Wozniak (2018)*.

## Studying mindfulness with neuroimaging using MRI—Concepts and technical aspects

In the early 1990s, functional magnetic resonance imaging (fMRI) made it possible to extensively and non-invasively study cerebral physiology and mental processes, which previously could only be investigated through joint analysis of lesions and disease. The physiological principal underlying fMRI derives from the fact that the magnetic properties of hemoglobin depend on the level of oxygenation in the brain: the BOLD effect (Logothetis and Pfeuffer, 2004). When activated by, e.g., a motor task, oxygen concentration in the related capillary network of stimulated cortical and subcortical areas exceeds normal levels. Brain activation can be compared during active performance and non-performance of a task because fMRI shows blood supply changes in the regions implicated in that task. A time series of individual fMRI scans extend over the course of minutes as the subject cycles through task and control conditions. Most commonly, analyses use a general linear model to make categorical comparisons of the conditions (Friston et al., 1995; Calhoun et al., 2001).

In regions not implicated in a task, we expect brain activity to decrease because sensory modalities are not stimulated (Haxby et al., 1994; Kawashima et al., 1994; Buckner et al., 1996). Deactivation should also be apparent in the frontal and posterior midline cortices (Ghatan et al., 1995; Baker et al., 1996). Andreasen et al. (1995) showed that these areas activated in a memory task and suggested that they were associated with personal reflection. Subsequently, Shulman et al. (1997) and Mazoyer et al. (2001) identified specific brain areas that were more active during passive than during goal-directed task conditions, constituting the “default mode network” (DMN). Gusnard and Raichle (2001) affirmed the functional importance of the passive resting state, proposing that it sustains a stable, unified representation of the individual in their environment: a self-representation. The discovery of the DMN provided significant impulse to explore human cognitive and psychological activity.

The DMN is explored with resting state-fMRI (rs-fMRI), a time series of individual scans taken over the course of minutes. Unlike act-fMRI, rs-fMRI are acquired only in the resting state and are not compared to scans taken in the active state (Biswal, 2012). Commonly, time correlations are computed among the regions of interest captured by brain images to establish functional connectivity. Regions that belong to the DMN are deactivated during act-fMRI studies but show increased activity during periods of reduced interaction with the external world, e.g., rest, sleep, or under anesthesia (Buckner et al., 2008). There is an anticorrelation between the DMN and externally activated networks. The DMN develop in early infancy and is deficient in Alzheimer's disease, autism, and schizophrenia (Buckner et al., 2008). Although we do not yet know its function, the components of DMN were revealed in studies of meditation, self-reflection, perception of prospects, and reflections about others during mentalizing, which is a form of cognitive empathy (Frith and Frith, 2003; Choi-Kain et al., 2008). These studies suggest clear differentiations between reasoning about another person's mental state and affective states shared with another person (empathy associated with distress) or concern for another (compassion), even though these behaviors interact under certain circumstances (Preckel et al., 2018). The two study paradigms, act-fMRI, and rs-fMRI, both acquire time series that extend over periods of minutes. Structural MRI (s-MRI) requires iteratively reconstructing k-space by acquiring signals averaged over minutes. s-MRI uses modern scanners to capture high resolution structural images. Researchers combined sophisticated analysis software with these high-resolution images to develop voxel-based morphometry, allowing them to measure the size of local gray matter in cross-sectional studies and tensor-based morphometry expressed by tensor gradients in longitudinal studies (Ashburner and Friston, 2000; Abela et al., 2014).

The three MRI study paradigms rely on segmenting brain matter into ventricles, white matter, and gray matter (the cortical layer and subcortical nuclei) and spatial standardization of individual brains. The creation of a common stereotactic space makes possible direct comparisons of regional changes in individuals or groups and allows us to study their relation to behavioral covariates. Long-term changes in local brain volumes over time may indicate brain plasticity, such as might be due to brain lesions or to physical or mental training (Debarnot et al., 2014).

A search of the MEDLINE metadata bank of references and abstracts on life sciences and biomedical topics (National Library of Medicine, USA) *via* PubMed yielded 12,008 publications on mindfulness since 1985 (Table 2) The Table shows the stepwise integration of various mental processes (e.g., meditation, awareness, believing, attention, mind wandering, and working memory) visualized with MRI into the research focus. This expansion of research focus concurred with the detection of the DMN.

**Table 2:** Literature search in PubMed [National Library of Medicine, USA].

**Assimilation of new settings into the context of mindfulness**				
**Keyword 1**	**Keyword 2**	**Keyword 3**	**Papers [*n*]**	**Since**
Mindfulness			12,008	1985
Mindfulness	Meditation		2,902	2001
Mindfulness	MRI		683	2001
Mindfulness	Meditation	MRI	127	2006
Mindfulness	Awareness	MRI	88	2000
Mindfulness	Believing	MRI	29	2001
Mindfulness	Focused attention	MRI	28	2003
Mindfulness	Mind wandering	MRI	28	2007
Mindfulness	Working memory	MRI	15	2005
Mindfulness	Open monitoring	MRI	4	2001

## Neuroimaging studies of mindfulness—Shaping the brain in parallel with the experience in mindfulness meditation

Using the neuroimaging techniques act-fMRI, rs-fMRI, and s-MRI, described in the previous section, we next present the results of selected original publications that discuss key elements of mindfulness and/or its behavioral covariates observed during naturalistic tasks (Gallagher and Brøsted Sørensen, 2006). Selection was performed with “mindfulness,” “focused attention (FA),” “open monitoring (OM),” “mind wandering,” “emotional regulation,” “magnetic resonance imaging (MRI)” and “default mode network (DMN).” We accommodated our approach to the suggestions of Gallagher and Brøsted Sørensen (2006) to reduce the data and extract the essentials from the observed behavior including its context and, thus, associate the core of the experiential phenomenology with the neuroimaging findings for objectivation. In essence, we categorized first-hand experience of phenomena at a level of abstraction sufficient to allow us to recognize the common properties of phenomenological data and objective data accepted by the sciences. These behavioral categories included task description, task performance, context of the task, explicit or implicit information processing, and experience in mindfulness meditation.

Researchers have characterized subjects' abilities to process emotions during different stages of meditation experience and while exposed to different conditions. Herwig et al. (2010)'s act-fMRI study of meditation-naïve healthy volunteers revealed that BOLD increased in the dorsal mPFC, extending to the superior frontal gyrus, during self-related perception and emotional introspection. At the same time, activity decreased exclusively in the left amygdala during emotional introspection. The unique association of BOLD responses in dorsal mPFC and amygdala during emotional introspection indicated that the phenomenon was independent of voluntary intention. BOLD response within the anterior mPFC during cognitive self-reflection and within the posterior mPFC during emotional introspection correlated inversely with FMI-scale score (Walach et al., 2006), which suggests that subjects with higher mindfulness scores use fewer neural resources. Application of the same study protocol confirmed that emotion-introspection downregulated amygdala activity in depressed patients, supporting its use as mindfulness related treatment (Herwig et al., 2018).

Murakami et al. (2015) continued to explore the relationship between the amygdala and the PFC in a study of unselected healthy subjects presented with images containing emotionally negative content. Subjects used two strategies, voluntary suppression and mindful emotional self-regulation, to cope with these negative images; both strategies reduced negative affect more than natural responses. These strategies suppressed the response of the amygdala, but act-fMRI suggested they each involved different neural systems. During mindful self-regulation, functional connectivity between amygdala and mPFC was prevalent; during voluntary suppression, functional connectivity between amygdala and dorsal lateral prefrontal cortex (lPFC) was prevalent. A post-examination interview indicated that mindful introspection was accompanied by more reliable self-monitoring. Kral et al. (2018) showed that amygdala response to negative emotional stimuli decreased in more experienced meditators, while short-term training in a mindfulness-based stress reduction course did not have the same effect. In an act-fMRI study by Lebois et al. (2015), healthy subjects who had not previously meditated were taught two strategies for disengaging from one sentence scenarios (i.e., stressful vs. non-stressful) projected on a screen. The first strategy was mindful attention (a decentring attitude) and the second was immersion in the scenario. Those who paid mindful attention showed less neural activity in the subgenual ACC, ventral ACC, ventral mPFC and medial orbito-frontal cortex during exposure to stressful scenarios that those who immersed themselves in the scenario. Three day intensive mindfulness meditation training intervention has been effective in reversing resting state functional connectivity between amygdala and subgenual ACC which associates previously perceived stress (Taren et al., 2014).

Reappraisal and acceptance can also be used to cope with emotionally negative experiences. Unlike acceptance, reappraisal is an elaborate cognitive practice in which one iteratively reinterprets negative experiences so that they eventually cease eliciting negative affect (Gross, 2001). In healthy subjects confronted with sad images, both strategies effectively regulated negative emotions better than no strategy; reappraisal was more effective than acceptance (Smoski et al., 2015). This act-fMRI study found the right frontal pole and medial frontal cortex were activated during acceptance. The left insula and precentral gyrus were activated during reappraisal. Opialla et al. (2015) observed activation in the left ventral and dorsal lPFC, supramarginal gyrus, and insula increased with mindfulness more than with cognitive re-appraisal during cued expectation of negative stimuli, but not during perception. Thus, initiation of a mindful state may engage more neural resources, specifically in the expectation phase of meditation-naïve subjects. Subjects who had major depressive disorders had less activity in their ventral medial PFC during mindful acceptance, a predictive sign of depressive relapse, and less activity in cognitive control regions like the paracingulate area, which may influence the ability to adapt emotional responses (Shackman et al., 2011).

Modinos et al. (2010) used an act-fMRI study design to investigate healthy subjects and determine the relationship between mean activation in dorsal mPFC during reappraisal and mindfulness traits as determined by KIMS. Subjects were taught a reappraisal strategy and then confronted with neutral and negative images during a fMRI task. Their dorsal mPFC was activated when they successfully reappraised the negative image while their amygdala deactivated. The positive association of mean activation in dorsal mPFC with mindfulness traits according to KIMS was dependent mainly on the subscale “act with awareness.” Other studies unrelated to mindfulness found that the degree of ventral mPFC activation was positively related to self-referential evaluations (Gusnard and Raichle, 2001; Northoff and Bermpohl, 2004) and evaluations of emotional stimuli affecting others (Frith and Frith, 2003).

Some effects of meditation practice were touched on above, and these and other effects have been the subject of extensive investigations. In an act-fMRI study, Farb et al. (2007) used a word perception task to show that experienced meditators were more likely to enter a state of experiential self-awareness than naïve subjects. Experienced subjects were trained daily in an 8-week mindfulness-based stress reduction course. They learned to discriminate between experiential and narrative forms of self-awareness, while untrained naïve subjects could not differentiate the two and tended to the narrative mode. All subjects were asked to engage in self-awareness while they were briefly shown a validated, randomized list of words chosen to elicit either positive or negative emotions (Anderson, 1968). BOLD response in both the ventral and dorsal mPFC markedly decreased in experienced subjects in the experiential mode. At the same time, activation shifted from midline structures to right prefrontal-lateral areas and the anterior insular cortex (AIC). The authors proposed this shift might be caused by consolidation of a decentred attitude and diminished self-referential neural processing. Farb et al. (2013) extended this study, employing the same subjects but changing the test paradigm. The revised study design included three conditions: interoceptive attention; word perception while refraining from cognitive or emotional response; and recognition of word repetitions. The state of interoceptive attention in experienced meditators deactivated the dorsal mPFC and activated the right posterior insula. Concurrent activation of the posterior and anterior insular cortex also indicated that the internal insular structure was reorganized. This activity pattern, including dorsal mPFC, might indicate the substratum in which tonic activity is preserved in the AIC during externally focused attention. The greater functional connectivity between posterior and anterior insula may enable subjects to integrate simultaneously interoceptive and exteroceptive processing. The crucial active involvement of the AIC during interoception and its importance for interoceptive accuracy, e.g., toward sensing the breathing rhythm, were established in recent fMRI experiments by Wang et al. (2019). Recently (Lenhart et al., 2020) reported findings similar to those of Farb et al. (2013) in a longitudinal study of gray matter changes in the course of a 7 weeks FA meditation training. They found increases in the AIC, the caudate nucleus and the frontal cortices, decreases in the parieto-temporal areas and the parahippocampal gyrus (PHG) as well as fractional anisotropy alterations adjacent to right hippocampus (HIC) and basal ganglia. Most important are the contributions of Santarnecchi et al. (2021) using rs-fMRI and determining mindfulness induced functional connectivity in the right putamen, cerebellum and anterior insula after an 8 week MBSR training. The prominent findings were the effective connectivity patterns between ACC, putamen on both sides and right cerebellum and the differential response of executive and somatosensory putaminal subregions within this network, exerting a modulatory functional impact both on orbitofrontal cortex and cerebellum.

A four-condition act-fMRI by Brewer et al. (2011) revealed that the activation pattern of experienced mediators was independent of three test conditions: attention to respiration; loving kindness; and neutral awareness. In contrast to controls deactivation of the posterior cingulate cortex (PCC) and mPFC, two constituents of the DMN, were observed in experienced meditators as part of this network during the three test conditions relative to baseline. Experienced mediators also exhibited more connectivity in PCC and areas involved in conflict monitoring, cognitive control and working memory (the dorsal lPFC and ACC) under all test conditions (Mansouri et al., 2009). Hasenkamp et al. (2012) and (Hasenkamp and Barsalou, 2012) used rs-fMRI to monitor cognitive processes in experienced meditators and a control group of subjects with little meditative experience. The authors differentiated four consecutive phases of a naturalistic cycle during self-monitoring: mind wandering, awareness of mind wandering, attention shifting, and re-established sustained attention. These form a theoretical model of cognitive fluctuations during a FA session. Experienced meditators were distinguished by diminished activity in a cluster that involved ventral mPFC and ACC when they shifted and restored sustained attention. Dorsal lPFC plays a key role in preserving FA (Hasenkamp et al., 2012). Significant differences between experienced and inexperienced mediators have also emerged in an analysis of functional connectivity. This analysis delineated a salient network involving DMN at ventral mPFC and bilateral PCC during mind wandering and involving executive network at dosal lPFC while sustained attention to breathing was restored. When experienced mediators paid sustained attention, connections between the right anterior insula, left dorsal IPFC, mid cingulate gyrus and right dorsal IPFC increased as did connections between a PFC/ACC cluster and bilateral inferior parietal lobules, while connectivity between a ventral mPFC/ACC cluster and the left PCC decreased (Hasenkamp and Barsalou, 2012). Similarly, in a rs-fMRI study of subjects who participated in a 4-day intensive meditation course resulting in sustained resilience for 3 months in contrast to controls in a relaxation retreat, Kwak et al. (2019) observed an increase in resting state functional connectivity between the dorsal mPFC and rostral ACC. A rs-fMRI study of healthy elderly subjects revealed that MAAS-scores and two constituents of the DMN (PCC and precuneus) correlated positively (Shaurya Prakash et al., 2013). The dorsal area of the PCC delineated most prominently in the correlation may interface between the resting state network and the network regulating cognitive control (Leech et al., 2011). The precuneus fulfills multiple functions, among them maintaining open monitoring (Gusnard et al., 2001).

The mind wandering phase of the monitoring cycle complements mindfulness. In an act-fMRI study of healthy volunteers, Mason et al. (2007a) explored the occurrence of stimulus independent thoughts (SIT) during verbal and visuo-spatial tests of working memory. They found a voxel-wise correlation between the tendency to daydream (assessed on the Imaginal Process Inventory-scale) (Singer and Antrobus, 1970) and constituents of the resting state network, e.g., the ventral and dorsal mPFC, posterior cingulate cortex and precuneus. They suggested as SIT accumulates during the tasks, the transition to mind wandering is inevitable. Mind wandering hinders mindful perception and eventually elicits ruminations about past and future. In an fMRI study of neural recruitment in both the default mode and executive networks, Christoff et al. (2009) suggested that mind wandering is most pronounced when meta-awareness is absent. Based on rs-fMRI, Wang et al. (2014) defined eleven nodes of the DMN based on their positive functional connectivity to PCC: PCC (1); mPFC (2); superior frontal gryrus on both sides (3, 4); lateral parietal cortex on both sides, LPC (5, 6) (i.e., BA 39); lateral temporal cortex on both sides, LTC (7, 8); PHG on both sides (9, 10); and thalamus, TH (11). Based on MAAS-scores, the link between thalamus and PCC most closely correlated with mindfulness. Nodal properties of the thalamus exhibited weak but significant negative correlations with these scores.

Mindfulness both activates distinct regions of the brain and induces morphological plasticity in the long-term. Using high resolution s-MRI, Murakami et al. (2012) established a correlation between the FFMQ-scale and the volume of the right insula and amygdala in healthy subjects. They suggest that volume increases in these structures might comprise a module in which the right insula facilitates physical interoception and the amygdala facilitates emotional response. Chronic emotional stress might also increase the volume of the amygdala, as Gianaros et al. (2008) observed. In a second s-MRI study of 247 college students with no previous experience of meditation, Lu et al. (2014) found MAAS scores correlated with the volume of gray matter in areas of the DMN and attention networks. The PCC on both sides, the left orbito-frontal cortices (OFC) and the right HIC/amygdala correlated negatively, while the dorsal ACC on both sides correlated positively. The positive correlation between MAAS score and dorsal ACC volume indicates that the dorsal ACC plays a role in sustaining attention and thus in conscious awareness. The negative correlation between MAAS score and PCC volume is consistent with the decrease in self-related thinking in more mindful students and the negative correlation between MAAS score and OFC and HIC/amygdala volume is consistent with reduced emotional responsiveness. In a third s-MRI publication, Zhuang et al. (2017) explored the disposition to mindfulness in a large group of young adults with no experience meditating. The authors connected MAAS and FFMQ scores to brain volumes and surface areas and found MAAS scores and gray matter volumes significantly correlated with the volume of the right precuneus, the surface area of the right dorsal lPFC (Brodmann area, BA, 46), the right inferior parietal lobule, IPL (BA40), and the left superior prefrontal cortex (BA 9). They also significantly correlated with the FFMQ items that comprised the category “describing.” These findings are consistent with increased self-awareness in more mindful young adults.

Several studies examined the effects of long-term meditation experience on brain morphology and function. Lazar et al. (2005) found focused attention significantly increased thickness in the right anterior insula and prefrontal cortex (BA 9 and 10); increases were less significant in the somatosensory, auditory, and visual cortices. Luders et al. (2012)'s cross-sectional study compared experienced meditators (mean ± SD 19.8 ± 11.4 years of experience) to healthy controls and found that in the experienced group mean curvature increased, suggesting increased cortical gyrification, maximal in the right anterior dorsal insula. Their prediction was based on the hypothesis that group differences and/or correlations would be most pronounced in cortical regions known to increase in volume in meditators, e.g., in the right anterior insula (Lazar et al., 2005; Hölzel et al., 2008). They also found that curvatures increased to a lesser extent in the left anterior dorsal insula, left precentral gyrus, right fusiform gyrus, and right cuneus. The authors emphasized the key role the anterior insula plays in long-term meditation. In a complementary rs-fMRI study, Taylor et al. (2013) found that the functional correlation between right IPL (BA 39) and dorsal mPFC (BA 10) was stronger in experienced mediators than in novices. This pattern of interconnected nodes suggests that long-term meditation may improve global attention rather than mindfulness, specifically since the parietal cortex is involved in working memory and affects visuo-spatial performance (Courtney et al., 1996). Recently, Fujino et al. (2018) discovered specific subcortical—cortical interactions in experienced meditators. Functional connectivity from the striatum to the posterior cingulate cortex diminished during FA meditation and during OM meditation, but functional connectivity from the ventral striatum to the retrosplenial cortex, which maintains memory function in the DMN, diminished only during OM-meditation. For the first time, this segregation from memory function was substantiated with neuroimaging, revealing a mechanism of detachment from self-relational thoughts.

When they compared different forms of meditation to visual stimulation in long-term meditators using fMRI, Josipovic et al. (2011) found anticorrelation between the task-positive extrinsic (the visual system) and the task-negative intrinsic (the DMN system) decreased during non-dual awareness (NDA) in the Tibetan tradition when referencing to anticorrelation observed in FA meditation. Most important, they found no differences between conditions in the modulation of brain activity in either network. NDA likely differs conceptually from FA and OM meditation, since NDA is context-oriented and FA and OM meditation are content-oriented (Josipovic, 2014).

**Figure 1:**
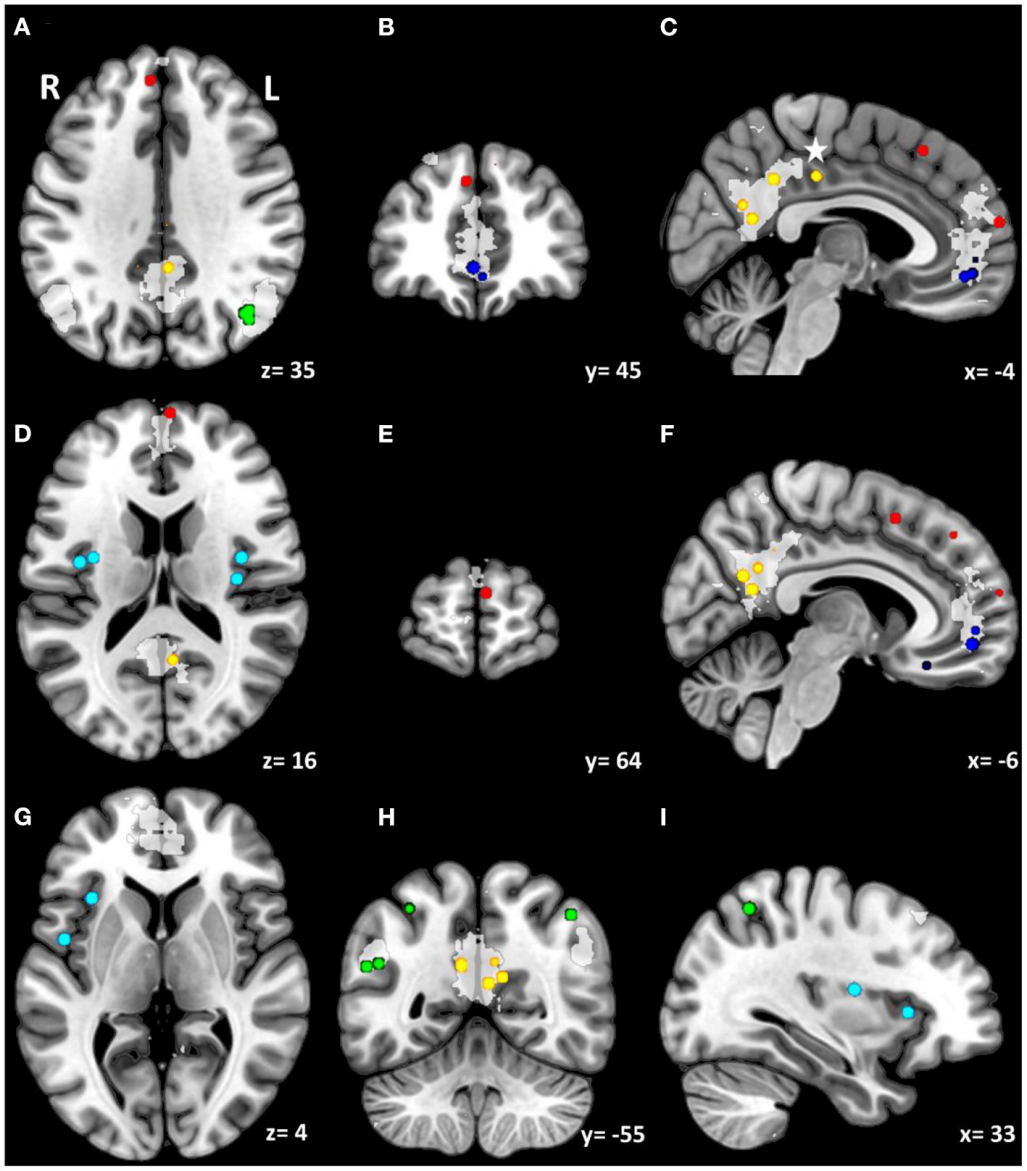
Involvement of default mode network constituents as well as the insular cortex. The Figure indicates centers of gravity of cortical involvement observed in selected neuroimaging studies detailed in Supplementary Table S3, integrated into the standard MNI 152 template. For according MNI coordinates see Supplementary Table S4. Enclosed is furthermore an automated topic-based meta-analysis using the term “DMN” in article abstracts provided by https://neurosynth.org/analyses/terms/dmn/ for comparison purposes. The light-gray areas superposed on the anatomical slices delineate zones preferentially associated with the term in 366 neuroimaging studies with an expected FDR <0.01. The red and dark blue spheres indicate the involvement of dorsal and ventral mPFC, and the yellow spheres indicate PCC involvement. **(A–F)** Self-specifying processes involve the dorsal mPFC whereas self-relational processes involve ventral mPFC, together with pre- and subgenual ACC, as well as PCC and retrosplenial cortex. Please note: Involvement of mPFC at the level of superior frontal gyrus is predominant in subjects of no meditation experience suggesting voluntary effort during a task. **(C,F)** The dorsal area of the PCC, marked by a star, may be a separate compartment: an interface between the resting state network and the network regulating cognitive control (Leech et al., 2011). Ad insular cortex (light blue): Proximal insular cortex is a primary interoceptive center with distinct homeostatic functions **(D,G,I)**, whereas dorsal anterior insular cortex has been shown to support explicit interoceptive attention **(I)** (Wang et al., 2019). **(A,H,I)** IPL (green spheres) at its posterior part (angular gyrus) is related to the DMN, whereas at its anterior part (supramarginal gyrus) to the FPCN.

Table 3 provides a summary of behavioral domains appropriate to the time dependent expertise in mindfulness experience, the observed neurophenomenology in the according tasks and the putative areas associated with the observed neurophenomenology. Figure 1 shows the center of gravity of these areas involved as they relate to DMN (dorsal and ventral mPFC, PCC, IPL) and the insular cortex. The underlying individual studies and the associated areas involved within DMN and insular cortex are summarized in Supplementary Tables S3, S4.

**Table 3:** The self-specifying process in mindfulness from subjects naïve in mindfulness to expert status proficient in the metacognitive skill.

**Observed neurophenomenology is dependent on duration of mindfulness training**				
**Status**	**Duration of meditation training**	**Behavioral domain**	**Neurophenomenology of tasks**	**Putative structures related to neurophenomenology**
No experience and novices	0	Mind wandering	Activity independent thoughts	FC bw mPFC, PCC and precuneus^a^
	On a waitlist for meditation courses	Emotion control	Voluntary suppression	Amygdala connected to dlPFC^b^
		Emotion control	Mindful self-regulation	Amygdala connected to dmPFC^b^
↓		Emotion control	Introspection vs. self-reflection	SLF (dmPFC) ↑ and amygdala ↓^c^
		Mindful disposition Mindful disposition	Expression of mindfulness traits according validated scales	FC bw PCC and Precuneus^d^ FC bw thalamus and PCC (structure)^e^
		Focused attention	“Distracted” awareness^*^	BOLD in DMN (↓), mostly in dmPFC ↓^f^
Initial experience	Months	Open monitoring	Experiential vs. narrative focus	dorsal mPFC ↓, ventral mPFC ↓^g^
↓		Open monitoring	Integration of IC and EC	dmPFC interacts across different conditions^g^
Advanced experience	Years	Open monitoring	Self-relational detachment	FC bw striatum and retrosplenial cortex^h^
		Steering mindful state	Recognition of lapse into MW	dorsal ACC, bilateral anterior insula ↑^i^
↓		Steering mindful state	Shifting from MW to FA	mPFC/ACC in high meditation practice ↓↓^j^
		Global attention	Moment-to-moment awareness	FC of dorsal mPFC L to IPL R ↑^k^
Experts	> 5 years	Focused attention	“Undistracted” awareness*	PCC ↓↓^l^
		Metacognition	Control across meditation states	Co-activation of mPFC, PCC, dorsal ACC, dlPFC^m^
		Non-dual awareness	Access to context-oriented info	Synergism between DMN and sensory networks, e.g., a visual network processing^n^

IC, interoception; EC, exteroception; FC, functional connectivity; MW, mind wandering; FA, focused attention; bw, between.

Appropriate references:

a Mason et al. (2007a);

b Murakami et al. (2015);

c Herwig et al. (2010);

d Shaurya Prakash et al. (2013);

e Wang et al. (2014);

f Scheibner et al. (2017);

g Farb et al. (2013);

h Fujino et al. (2018);

i Hasenkamp et al. (2012);

j Hasenkamp and Barsalou (2012);

k Taylor et al. (2013);

l Garrison et al. (2013);

m Brewer et al. (2011);

n Josipovic et al. (2011).

* “Distracted” awareness means switching between FA, MW and refocusing whereas “undistracted” awareness means longer phases of undisturbed focusing.

## Discussion: Deducing the neurobiological underpinnings of mindfulness from brain imaging—A conceptual approach

Specific brain areas reflect mindfulness cultivated in FA- and OM- meditation training. These areas reflect the degree of experience meditators acquire as they practice and they specifically involve the EES, EPS, NS and FPCN systems, cf (Vago and Silbersweig, 2012).

### Self-relational processes and the midline structures of hemispheres

Gallagher (2000) describes the implications for cognitive science, positing in his philosophical conceptions of the self that people have a minimal embodied self-representing consciousness as an immediate subject of experience, existing in the present. He also posits that people possess a supplementary narrative self—a self-image that includes a past and future, inherent in the stories they formulate about themselves. Kyselo (2014) commented on these two aspects of the self, emphasizing that social existence is organized in terms of back-and-forth between social distinction and participation processes. In their view, the body becomes the mediator of these processes. The subject's narrative relies on self-relational processing that includes implicit subjective feelings and explicit cognitive thoughts, each of which are mediated by a task-negative network of cortical midline structures (Northoff et al., 2006) that comprise the ventral-medial prefrontal cortex (ventral mPFC), pre- and subgenual ACC (preACC, sgACC), posterior cingulate cortex (PCC), retrosplenial cortex (RSP), PHG and HIC.

The activity in the ventral mPFC decreases when subjects psychologically distance themselves from self-representations. Inversely activity in the ventral mPFC increases when personal values and self-related thoughts are involved (D'Argembeau, 2013). The ventral mPFC allows subjects to incorporate the interests of the self into an episodic event of the past while the HIC ensures they subject can recall this even in detail (Kurczek et al., 2015). This discovery may help us understand future thinking: patients with ventral mPFC lesions could not describe events in their own future in any more detail than they could describe events that happened to other people in the past (Verfaellie et al., 2019).

Garrison et al. (2013) took a neurophenomenological approach to studying undistracted awareness and effortless doing, and found they were associated with PCC deactivation, while distracted awareness and controlling were associated with PCC activation. Coincident subjective experience during these two antagonistic mental conditions (meditative vs. self-related) were quite different and seemingly specific (Garrison et al., 2013). On the basis of its connections, it seems the RSP is uniquely positioned to translate between the world-centered domain, including perirhinal gyrus, HIC and PHG, and the self-centered world of the medial parietal cortex (Vann et al., 2009). These are the areas involved in the phases of mind wandering (Mason et al., 2007a; Hasenkamp and Barsalou, 2012).

Analogous mechanisms have been uncovered in the course of believing processes, displaying activation patterns while subjects evaluated self-related interests or preferences. Independent of testable and non-testable beliefs, main effects of certainty were evident in the involvement of a midline neuronal network encompassing the left mPFC at intermediate z-level, caudate and PCG, and right superior temporal lobe in the neighborhood of temporo-parietal junction (TPJ). Certainty of a non-testable proposition, a strong belief, activated the left insula (Howlett and Paulus, 2015). Common areas engaged in false belief reasoning and visual perspective taking, which is a precondition for assessing the perspective of another subject while mentalizing, are evident in the left angular gyrus; these areas include the temporo-parietal junction, and the left medial occipital gyrus (Schurz et al., 2013). Incongruent mental states (false beliefs and unfulfilled desires) and congruent mental states also significantly increase the involvement of PCC/RSC in processing unfulfilled desires, while the same level of involvement is not shown for true beliefs (Abraham et al., 2010).

### Unconscious self-specifying processes

EES integrates implicit activity of a subject with prevalent automatic responses to extero- and interoception (Aspell et al., 2013), functional also outside of the focus of awareness (Roeser and Peck, 2009). Enactivism is primarily an implicit ongoing iterative process that helps the subject create a world of meaning through interaction with the environment, including other subjects. Enactivism is independent of logic presumptions and does not rely on representations (Nehaniv et al., 2013; de Haan, 2021). The process of enactivism is supported by embodiment, which structurally couples the subject with the world and results in non-conscious embodied actions (Nehaniv et al., 2013; Izmirli, 2014; Varela et al., 2016). As detailed above, EES is linked with the NS by the midline brain structures but distinguished from the NS by the underlying task-positive network.

In contrast, active enactive experience involves the subcortical-level midbrain nuclei, superior colliculi, medio-dorsal and ventral-posterior thalamus, pulvinar and dorsal striatum, and the cortical level proximal insula, premotor, and sensory association areas (Damasio, 1999; Craig, 2003). Activation of the proximal insular cortex is prototypical for afferents that transmit physiological information about distinct homeostatic sensory modalities. Proximal insular cortex activation is associated with an equivalent homeostatic emotion that engenders distinct body feelings and preserves physiological balance (Craig, 2004). In humans, an increasing proximal-to mid-to anterior pattern parallels integration of distinct sensory information and contextual affective contents [Stephan et al., 2003; Bud Craig, 2009]. This homeostatic processing provides the subject with diverse information (homeostatic motor functions, and environmental, hedonic, motivational social and cognitive conditions) that is integrated into a meta-representation within AIC, and with simultaneous co-activation in the ACC. This information, merging from various sources into a meta-representation, creates an emotional moment characterized by a specific feeling and an associated emotion (Craig, 2009). Sterzer and Kleinschmidt (2010) discuss the role of AIC in perceptual processes, asking if the AIC supports awareness of the immediate moment in a state of a subject's heightened alertness. Farb et al. (2007) validated the active involvement of a right lateralized network including lPFC, AIC, secondary sensory cortex, and inferior parietal lobule, which suggests experiential focus centers on the present in trained meditators. Mindfulness meditators may perceive a slowing of time in the present based on their ability to focus more strongly on sensory experiences and to be more strongly aware of feelings and of body states (Wittmann and Schmidt, 2014). The argument that AIC is integrated into perception of time intervals in the range of seconds to sub-seconds is supported by fMRI task results (Livesey et al., 2007). In the context of this section the involvement of large-scale networks should be noted as reported in recent papers, including subcortical gray and white matter, brain stem and cerebellum (Tang et al., 2015; Lenhart et al., 2020; Santarnecchi et al., 2021).

### Conscious self-specifying processes

Arising from unconscious information processing of EES, subjects develop a self-as-subject or a minimal self that is not taken as an intentional object; instead, it acquires knowledge from a first person perspective (Gallagher, 2000; Legrand, 2007). At the level of the experiential phenomenological self, individuals consciously perceive information of subjective content, however these percepts cannot be transformed into third person data through traditionally valid scientific procedures (Gallagher and Brøsted Sørensen, 2006). To prevent methodological biases (Gallagher, 1997; Gallagher and Varela, 2003) suggested a framework of reflective, methodically guided phenomenological analysis of behavior to get information about the phenomenal self—the “me.” The task-positive mental processes of experiential self are associated with attention and anticorrelated to the task-negative mental processes of NS, which are associated with long-term memory (Buckner and Carroll, 2007).

The synopsis of conscious self-specifying vs. self-related processes together with functional and structural neuroimaging studies yields main findings as detailed in the section “Neuroimaging studies of mindfulness—shaping the brain in parallel with the experience in mindfulness meditation.” Functional connectivity between PCC and thalamus plays a dominant role in self-specifying processes. Connectivity is inversely proportional to mindfulness (Wang et al., 2014). Dorsal mPFC is a main hub of mindful disposition and behavior over a wide range of experience in meditation training (Farb et al., 2007, 2013; Herwig et al., 2010; Modinos et al., 2010; Kral et al., 2018). Specific strategies against negative emotions are clear. The right dorsal mPFC correlates with the left amygdala (Murakami et al., 2012) when viewers see pictures with negative valence; dispositional mindfulness correlates with mindful disposition, based on KIMS. When reappraisal for anticipating negative emotions was directly compared to voluntary suppression, the pathway for anticipating negative emotions was through the right dorsal mPFC to left amygdala, and the pathway for voluntary suppression was through the right dorsal lPFC and left precuneus to the left amygdala (Murakami et al., 2015).

The activation pattern in emerging daydreaming changes from the dorsal mPFC to the ventral mPFC and PCC when the thinker transitions to self-relational thoughts (Mason et al., 2007a). In elderly individuals, connectivity between posterior PCC and medial precuneus cortex correlates with mindful traits, which may reflect the multiple functions of PCC at this site, some of which may specifically maintain open monitoring (Gusnard et al., 2001; Shaurya Prakash et al., 2013). When ruminative thoughts simulated by immersion were compared to disengaging by subjective decentring, researchers found distinct spatial patterns in the structures involved for each condition in non-meditative individuals. Mental immersion involved brain areas that reflected bodily and experiential self-relation, e.g., ventral mPFC, mOFC, vACC, sgAC. Mindful intention involved areas that indicated perspective shifting, e.g., dorsal mPFC, IPL, including angular gyrus (Lebois et al., 2015).

In an experiential vs. narrative test paradigm, an 8-week mindful meditation training course reduced BOLD in the dorsal mPFC to levels lower than those found in meditation novices in Farb et al. (2007). Developing interoceptive attention and mindfulness training evoked greater activity in the anterior insula in experienced meditators (Craig, 2002; Farb et al., 2013). Hasenkamp et al. (2012) found the involvement of dorsal ACC and bilateral AI enhanced when the subject became aware of lapse into mind wandering. Hasenkamp and Barsalou (2012) found the involvement of ventral mPFC/orbitofrontal cortex diminished when the subject shifted from mind wandering to focused attention. In novices this switching between mindful attention, mind wandering and refocusing causes distracted awareness associated with diminished activities within constituents of DMN (Scheibner et al., 2017). Long-term meditators exhibit fundamentally functional changes in DMN connections (Taylor et al., 2013). Very experienced meditators achieved in a FA-task the level of one-point concentration providing them undistracted awareness associated with pronounced activity decrease in PCC (Garrison et al., 2013). Strong connections were evident between dorsal mPFC and R IPL (most likely corresponding to angular gyrus), precuneus/PCC and R IPL, and R IPL and L IPL (Taylor et al., 2013), which suggest enhanced functional working memory and attention, and diminished self-relational processing (Culham and Kanwisher, 2001; Northoff and Bermpohl, 2004; van Buuren et al., 2010). In masters of introspection, awareness and emotional control, the dorsal-anterior insula was the site of an increase in global maximum gyrification, suggesting this area plays a key role in integrating autonomic, affective, and cognitive processes (Luders et al., 2012). We can distinguish complex NDA meditation from FA and probably also OM meditation because extrinsic networks processing experiences related to the environment and intrinsic networks processing experiences related to interoception are increasingly synergistic in meditators proficient in NDA meditation than competitive (Josipovic, 2014).

### Learning processes

Meditation is a form of mental training to acquire the basic prerequisites for maintaining a mindful disposition. In a meta-analysis of 78 functional neuroimaging (fMRI and PET) studies (Fox et al., 2016) found specific but diverging patterns of activations and deactivations when comparing FA, mantra recitation, OM and compassion/loving kindness meditation. Peak activation likelihood estimate (ALE) was given in FA and OM meditation, we were focusing on according to selection criteria for the review: In FA peak values for activations involved left premotor cortex, supplementary motor area, right putamen/lateral globus pallidus, right fusiform gyrus, right cuneus and left precuneus, and peak value for deactivation left anterior insula; in OM peak values for activations involved right anterior insula, right parieto-occipital sulcus and right somatosensory cortices/inferior parietal lobule. In a recent brain theory of meditation (Raffone et al., 2019) suggest a left-brain dominance for top-down regulation in FA meditation and a predominant cognitive and emotional processing in right anterior areas such as the anterior insula connected with the homotopic left hemispheres *via* the frontal parts of corpus callosum. The authors differentiate the mechanism for optimized use of brain resources in FA and OM, through reduction of firing neurons in the former and through tuning the communication between widespread neurons with higher firing rates in a given time window in the latter. Hernández et al. (2018) delineated an ultimate goal of long-term meditators in Sahaja Yoga Meditation tradition: The capacity to maintain a state of mental silence was based on a larger gray matter volume in right anterior cingulate cortex/medial PFC, while performance during scanning evoked increased functional connectivity of this region with bilateral AIC, and decreased functional connectivity with right thalamus/PHG.

In the transition phase between unconscious and conscious processes, contemplative practices may foster attention, emotion regulation, and introspection. These practices may eventually cultivate the habitual patterns of thoughts and beliefs NS provides and establish a mindful disposition governed by the EES (Vago, 2014; Seitz et al., 2016). Technology-mediated mindful intervention studies that used electroencephalographic frequency data to provide the user with real-time acoustic feedback, provide preliminary evidence that mindfulness effectively promotes conscious access to implicit information (Balconi et al., 2017). The evidence from electrophysiological observations is striking. For example, in subjects practicing mindfulness, we see the amplitude of the late positive potential (LPP) decreased after only 400 ms after viewing negative images. Temporo-parietal positivity associated with identification, evaluation, and labeling of the visual stimulus occurs between 600 and 1,000 ms (Brown et al., 2013). Consistent with these findings, mindful intervention during initial observation of negative pictures induced an alternative pathway in which the dorsal mPFC was involved. Late voluntary suppression of the full-blown affect mainly involved the dorsal lPFC (Murakami et al., 2015). The predominant pattern in mindful intervention might illustrate a transition from conceptual to non-conceptual awareness, reducing habitual evaluative processing and involving other areas like the thalamus, insula, sensory, and motor regions (Craig, 2003; Farb et al., 2013). Increased conscious awareness at the somatic and mental level may couple the sensory system to the organism and the environment and at the social level provide more participation (Varela et al., 2016).

Self-specifying and self-relational processes involve the cortical midline structures of DMN in distinct and partly antagonistically manners. These divergences reflect different behavioral levels of concepts of the self (e.g., sensory processing, self-referential processing, higher order processing), which interact in both bottom-up and top-down directions (Northoff et al., 2006). These process dynamics and their mutable participation in mentation shape DMN compartments to cognitive and contextual domains and influence their interaction. This influence is reflected in the evolution of functional and structural cortical patterns in the continuum from subjects naïve in meditation to subjects with long-term meditation experience (Josipovic et al., 2011; Josipovic, 2014). Patterns may change over the lifespan. When (Crane et al., 2020) explored links between personality traits in older people and cognitive performance and the default mode network, they found open perception was associated with three nodes: mPFC; middle frontal gyrus; and dorsal PCC, which may correspond to area 7 m outside of the DMN proper (Vogt et al., 2006; Leech et al., 2011; Shaurya Prakash et al., 2013).

## Conclusions

Mindfulness is set by the immediate subject of experience, unextended in time. This is different from the narrative of individuals—a self-image with a past and a future. The immediacy of mindfulness initiates self-specifying processes, primarily at the unconscious level of enactivism and embodiment, and secondarily at the conscious level of experiential phenomenological awareness. Necessary pre-conditions are competence to pay sustained attention, detachment from self-relational thoughts and preferences, and a non-aversive attitude. Practiced over the long-term, mindfulness will improve individual and social wellbeing. As meta-cognitive skill it enables the subject to monitor perception and behavior.

Critical questions remain to be answered. Subjects of varying meditative experience exhibited considerably variable cortical sites of co-activations, so we must clarify the role the dorsal mPFC plays in mindful tasks within the extending area of the midline prefrontal cortex. We might be able to elucidate the exact structural and functional segregation of ventral mPFC by connecting activation likelihood estimates (ALE) of neuroimaging meta-data to specific behavioral paradigm classes of assigned tasks (Bzdok et al., 2013). We also need to understand how the diverse mindful traits are assigned to common or diverse neural networks, and to learn more about how mindfulness increases the capacity of a meditator's working memory (Jha et al., 2010; van Vugt and Jha, 2011; Mrazek et al., 2013). How is effortless attention differentiated from forceful cortical control mechanisms, especially when we perform demanding naturalistic tasks (Gallagher and Brøsted Sørensen, 2006; Jensen et al., 2012; Raffone et al., 2019)? We need to know why segregation of resting state networks seems to decrease processing speed in older subjects when constituents of fronto-parietal control network are affected (Malagurski et al., 2020). Finally, the modified resting-state in long-term meditators may affect mind wandering since the mindfulness may evolve in ways that alter in-parallel self-relational thoughts and induce a more positive mood (Vago and Zeidan, 2016).

Implementing the neuroimaging techniques s-MRI, act-fMRI, and especially rs-fMRI, was a major step forward. These techniques help us understand the dynamic processes underlying mindfulness, follow the process of learning the meta-cognitive skill of mindfulness from early to long-term experience in meditation, and delineate the governing structure of the DMN. The main constituents of DMN are the dorsal mPFC, ventral mPFC, and PCG, which differentially interact depending on the subject's experience in meditation. The midline structures of dorsal mPFC, ventral mPFC, and PCG are antagonistic to self-specifying and self-relational processes, so they allow approximate discrimination in-between. AIC is a meta-representation for sensory perception that integrates both interoception (the self-centered world) and external perception (the world-centered domain). Brain volume changes may indicate brain plasticity, mediated by mental training over the long-term.

## Data availability statement

The original contributions presented in the study are included in the article/Supplementary material, further inquiries can be directed to the corresponding author.

## Author contributions

BW conceived and wrote the systematic review, conducted the search, and screened the titles, abstracts, and full texts of the paper.

## Funding

This paper was funded by Dr. Rüdiger Seitz, via the Volkswagen Foundation, Siemens Healthineers, and the Betz Foundation. Siemens Healthineers was not involved in the study design, collection, analysis, interpretation of data, the writing of this article or the decision to submit it for publication.

## Conflict of interest

The author declares that the research was conducted in the absence of any commercial or financial relationships that could be construed as a potential conflict of interest.

## Publisher's note

All claims expressed in this article are solely those of the authors and do not necessarily represent those of their affiliated organizations, or those of the publisher, the editors and the reviewers. Any product that may be evaluated in this article, or claim that may be made by its manufacturer, is not guaranteed or endorsed by the publisher.

## Footnotes

^1^Dharamsala.

## Abbreviations

FA: Focused Attention
OM: Open monitoring
SIT: Stimulus Independent Thought
SOT: Stimulus Oriented Thought
MW: Mind Wandering
EES: Enactive Experiential Self
EPS: Experiential phenomenological Self
NS: Narrative Self
MRI: Magnetic Resonance Imaging
s-MRI: structural Magnetic Resonance Imaging
act-fMRI: functional Magnetic Resonance Imaging during activation task
rs-fMRI: functional Magnetic Resonance Imaging during resting state
BOLD: Blood Oxygen Level Dependent
DMN: Default Mode Network
FPCN: Fronto-parietal control network
mPFC: medial Prefrontal Cortex
lPFC: lateral Prefrontal Cortex
PCC: Posterior Cingulate Cortex
ACC: Anterior Cingulate Cortex
AIC: Anterior Insular Cortex
OFC: Orbitofrontal Cortex
IPL: Inferior Parietal Lobule
RSP Cortex: Retro-Splenial Cortex
PHG: Para-Hippocampal Gyrus
HIC: Hippocampus
TPJ: Temporo-parietal Junction
KIMS: Kentucky Inventory of Mindfulness Skills
MAAS: Mindful attention Awareness Scale
FMI: Freiburg Mindfulness Inventory
FFMQ: Five Facet Mindfulness Questionnaire.
