# Supplementary material for: Mindfulness in the focus of the neurosciences - The contribution of neuroimaging to the understanding of mindfulness
Source: Front Behav Neurosci. 2022 Oct 17;16:928522. doi: 10.3389/fnbeh.2022.928522 (PMC9622333; doi:10.3389/fnbeh.2022.928522)
Supplement: Supplementary file 1 [file Data_Sheet_1.pdf]

Supplementary Material

Mindfulness in the focus of the neurosciences -

The contribution of neuroimaging to the understanding of mindfulness

Bruno J. Weder

Table ST1. Paper selection for the review

Table ST2. Scales measuring mindful traits and tendency to lapse into mind wandering

Table ST3. Synopsis of selected studies dealing with mindfulness

Table ST4. Coordinates of DMN constituents and insular zones involved - Supplementary Table for Figure 1

Table ST1. Paper selection for the review

Search terms for selection of papers to include into the sections 2 to 6 of the paper which represent the basis for the final discussion "Deducing the neurobiological underpinnings of mindfulness from brain imaging – a conceptual approach"

<u>Section 2. The quest for an operational definition of mindfulness – a semantic issue</u>

Core elements of mindfulness, collateral features

Training, focused attention (FA) meditation, open monitoring (OM) meditation

Self-referential thinking, mind wandering, processes competing with mindfulness

Excluded: meditation forms beyond FA meditation and OM meditation

<u>Section 3. Dimensions of mindfulness</u>

First person perspective, the subject of experience, the metaphysical self, the "I"

Third person perspective, the phenomenal self, the "me"

Self-specifying processes, self-relational processes

Conceptual frameworks, relationship between mindfulness processing and neurobiological mechanisms

Attentional expertise, concentration meditation

<u>Section 4. Dimensions of believing and interrelations with mindfulness</u>

Believing as an example of self-referential processing

Interaction of mindfulness with believing

Competition of believing with mindfulness

<u>5. Studying mindfulness with neuroimaging using MRI – concepts and technical aspects</u>

Examining mindfulness using structural MRI, functional MRI

Resting state functional MRI

Default mode network, functional characteristics

Voxel-based morphometry, tensor-based morphometry

<u>6. Neuroimaging studies of mindfulness – shaping the brain in parallel with the experience in mindfulness meditation</u>

Naïve in meditation, experience in meditation, long-term meditation

Tasks for mindfulness, Tasks for mind wandering

Brain plasticity, experience in meditation, long-term meditation

Differentiated involvement of DMN, emotional control, mindful processing, narrative processing

Interoception, exteroception, insular cortex

Effortless attention, cortical control mechanisms

Long-term effects and working memory, and mind wandering

Table ST2. Scales measuring mindful traits and tendency to lapse into mind wandering

1. The Mindful attention Awareness Scale [MAAS] comprises 15 items related to daily self-perception and -control that express the single factor, mindfulness (Brown & Ryan, 2006). To focus on fundamental aspects, items containing attitudinal components like acceptance, motivational intent and potential consequences like calmness were excluded. The scores range between 1 and 6. Although they converged to those of various assessments of well-being, the correlations were at best moderate.
2. The Freiburg Mindfulness Inventory [FMI] comprises 30 items covering a common scale of mindfulness for individuals acquainted with meditation or 14 items in an abbreviated version that do not assume Buddhistic or meditative practice (Walach et al., 2006). The scores range between 1 and 4.
3. The Kentucky Inventory of Mindfulness Skills [KIMS] is a 12 item scale dealing with 4 factors: describe, observe, act with awareness and accept without judgement(Baer et al., 2004). The scores range between 1 and 5.
4. The Five Facet Mindfulness Questionnaire [FFMQ] merges items from various scales into 5 subscales describing: non-reactivity, 7 items; observing, 8 items; acting mindfully, 8 items; describing mindfulness, 8 items; and non-judging, 8 items (Baer et al., 2006). The scores range between 1 and 5. A discrepancy between these scales and the two component model of mindfulness was pointed out by Malinowski (Malinowski, 2008). Three out of the five items, e.g. “acting mindfully”, “describing mindfulness” and “non-reactivity” might entail semantic problems due to their dependence on the meditative experience of the examinee, i.e. control subjects, novices or masters might understand the items differently.
5. The Imaginal Processes Inventory comprises questions regarding concrete situations that favour lapse into day dreams or mind wandering. Part I, an abbreviated form, poses 24 questions, Part II, a comprehensive form, poses an additional 320 questions. The scores range between 1 and 5. The test subjects are instructed explicitly not to ponder the perspective of the situation.

Table ST3. Synopsis of selected studies dealing with mindfulness

Authors	*Goup size [n]*	*Age [yrs]*	*Experience in meditation.*[1]	*Task*	*Context of the task*	*Mainly involved areas, MNI-coordinates*
1.Herwig et al. 2010	30	23 - 41	0	act-fMRI	Introspection of emotion	SFG R (15,-10,57)<, AMG L (-26,-7,-15)>
2.Murakami et al. 2015	21	20 -41	0	act-fMRI	Emotion regulation vs suppression	dmPFC R (6,46,36) corr. to AMG L
3.Modinos et al. 2012	18	20 -41	0	act-fMRI	Re-appraisal of neg. emotions	dmPFC R,L (6,34,54;-8,42,54) pos. corr. to KIMS
4.Mason et al. 2007	19	not known	0	rs-fMRI	Daydreaming evoked by SIT	vmPFC L (-6,51 -9)<, PCC/Precuneus(2,44,-6)<
5.Wang et al. 2014	245	21.5(±1.5 s.d.)	0	s-MRI	Bias to mind wandering	High connectivity between thalamus and PCC (6,-46,22)
6.Murakami et al. 2012	19	18 - 24	0	s-MRI	+ corr. to describing item (FFQM)	GM volume of AIC R(37,10,-15) and AMG L(13,-7,-13)
7.Lu et al. 2014	247	19 – 25	0	s-MRI	- corr. to mindful traits (MAAS)	GM volume of PCC R,L(12,-44,38) and L OFC(-40,36,-4)
8.Prakash et al. 2013	25	60 - 75	0	rs-fMRI	+ corr. to mindful traits (MAAS)	FC dorsal PCC (-2,-24,38) to Precuneus(8,-56,36)
9.Lebois et al. 2015	30	18 - 25	0	act-fMRI	Immersion>mindful attention	vmPFC/OFC(0,24,-12) <
					Mindful attention>immersion	SFG/dmPFC L (-15,9,57)<, Angular G. L (-48,-57,39) <
10.Wang et al., 2018	44	19-29	0	act-fMRI	IC attention > EC attention	AIC R,L (34,20,4 and -30,20,8) <
11.Farb et al. 2007	16	45.6(±13.4 s.d.)	+ vs ++	act-fMRI	Experiential vs narrative focus	dmPFC(-12,56,40) >,vmPFC(8,68,8) > in MT-group
12.Farb et al. 2012	16	45.6(±13.4 s.d.)	+ vs ++	act-fMRI	Intero- vs. exteroceptive attention	dmPFC (-3,27,51) interacts across conditions in MT-group
13.Hasenkamp et al. 2012	24	28 – 66	+++	act-fMRI	Cycle from FA to MW to	dmPFC (1,41,14) > across conditions
14.Hasenkamp and B. 2012	24	28 - 66	+++	act-fMRI	Awareness to Shift back to MW	dlPFC R(42 32 34) during FA > during MW
15.Taylor et al. 2013	13	46(±11 s.d.)	+ vs +++	rs-fMRI	Present-moment awareness	FC of dorsal mPFC(-10,57,19) to IPL(48,-56,27) rt <
16.Brewer et al. 2011	12	not known	++++	act-fMRI	FA , choiceless awareness,	mPFC/ACC (-6,52,-2) > and PCC(-8-56,26) >
					loving kindness	Co-activation of mPFC,PCC,dACC and dlPFC across meditations
17.Luders et al. 2012	50	24 - 71	++++	s-MRI	Long-term meditators	Gyrification in AIC<<
18.Josipovic et al. 2012	22	32 – 62	++++	act-fMRI	FA- versus NDA-meditation	Modulation bw. ex- and intrinsic system of the brain

Legend: sMRI, structural MRI; rs- fMRI, functional MRI during resting state; act-fMRI, functional MRI during activation task.
[1] Experience in meditation: none, 0; novices, +; > 2 month of training, ++; >1 year of training +++; > 5 years of training, ++++; dm dorsal medial, vm ventral medial, dl dorsal lateral
SFG, Sup. Frontal Gyrus; dorsal or ventral mPFC, dorsal or ventral medial prefrontal cortex; dl PFC, dorsal lateral prefrontal cortex; GM, grey matter; PCC, posterior Cingulate Cortex; AIC, anterior Insular cortex; OFC, Orbito-Frontal Cortex; dACC, dorsal Anterior Cingulate Cortex; AMG, Amygdala; FA, Focused attention; MW, Mind wandering; SIT, stimulus induced thoughts; MT, Meditation; FFQM, MAAS, KIMS, see Mindfulness Scales; <, increase, > decrease, corr., correlation; FC, functional connectivity; IC, interoceptive; EC, exteroceptive

Table ST4. Coordinates of DMN constituents and insular zones involved - supplementary Table for Figure 1

	dmPFC			vmPFC			PCC/ Precuneus		
	x	y	z	x	y	z	x	y	z
Herwig et al.,2016, feel>think	15	-10	57						
Herwig et al.,2016, think>feel	-9	14	52				-5	-55	18
Murakami,2015,observe(emotion)	6	46	36						
Murakami,2015, suppress(emotion)							-6	60	24
Modinos,2012,Re-appraisal	R 6/L -8	R 34/L 42	R 54/L 44						
Modinos,2012, Re-appraisal									
Mason et al.,2007, MW				-6	51	-9	-3	-45	37
Wang et al.,2014, Bias to MW				2	44	-6	6	-46	22
Murakami et al.,2012, corr.to FFQM									
Lu et al.,2014, corr. to MAAS							12	-44	38
Prakash et al., 2013, corr. to MAAS							-2	-24	38
Lebois et al.,2015, immersion				0	24	-12			
Lebois et al.,2015, mindful attention	-15	9	57						
Wang et al. 2019, IC vs EC									
Farb et al., 2007, EF vs. NF(novives)	-4	64	16				-8	-52	28
Farb et al., 2007, EF vs. NF(MT)	-12	56	40	8	68	8			
Farb et al. 2012, interoception (MT)							-12	-54	21
Farb et al. 2012, exteroception (MT)	-3	27	51						
Hasenkamp et al., 2012, Shifting MW to FA				1	32	-10			
Hasenkamp & Barsalou,2012, maintaining FA				-9	29	-19			
Taylor et al. 2013, to present moment aware	-10	57	19	-2	47	-10	8	-53	27
Brewer et al., 2011, three meditation tasks				-6	52	-2	8	-56	26
Josipovic, 2012				2	51	10	0	-31	29

Table S4 - continued

	Insula			IPL		
	x	y	z	x	y	z
Herwig et al.,2016, feel>think						
Herwig et al.,2016, think>feel						
Murakami,2015,observe(emotion)	-38	-6	16			
Murakami,2015, suppress(emotion)				-42	-66	36
Modinos,2012,Re-appraisal				R 54/L -42	R -54/L -66	R 26/L 36
Modinos,2012,Re-appraisal	R 47/L-36	R 0/L -16	R 4/L 17			
Mason et al.,2007, MW						
Wang et al.,2014, Bias to MW				R 48/L -42	R -60/L -70	R 30/L 34
Murakami et al.,2012, corr.to FFQM	37	-10	-15			
Lu et al.,2014, corr. to MAAS						
Prakash et al., 2013, corr. To MAAS						
Lebois et al.,2015, immersion				-45	-54	51
Lebois et al.,2015,mindful attention	R 34/L -30	R 20/L 20	R 4/L 8			
Wang et al. 2019, IC vs EC						
Farb et al., 2007, EF vs. NF (novices)	40	-8	16			
Farb et al., 2007, EF vs. NF (MT)	33	-6	15			
Farb et al. 2012, interoception (MT)				33	-57	54
Farb et al. 2012, exteroception (MT)						
Hasenkamp et al., 2012, Shifting MW to FA						
Hasenkamp & Barsalou, 2012, maintaining FA				R 48/L -40	R -56/L -67	R 27/L 34
Taylor et al. 2013, to pesent moment aware				54	-36	45
Brewer et al., 2011, three meditation tasks						
Josipovic, 2012						

Legend: FA, Focused attention; MW, Mind wandering; SIT; MT, experienced in meditation; FFQM, MAAS, see Mindfulness Scales; corr., correlation; EF, experiential focus; NF, narrative focus; R, right; L, left; dmPFC, dorsal medial Prefrontal Cortex; vmPFC, ventral medial Prefrontal Cortex, PCC, Posterior Cingulate Cortex; IPL, Inferior Posterior Lobule
